# Biomaterials targeting the microenvironment for spinal cord injury repair: progression and perspectives

**DOI:** 10.3389/fncel.2024.1362494

**Published:** 2024-05-09

**Authors:** Yating Gao, Yu Wang, Yaqi Wu, Shengwen Liu

**Affiliations:** ^1^Department of Neurosurgery, Tianyou Hospital, Wuhan University of Science and Technology, Wuhan, China; ^2^Department of Neurosurgery, Tongji Hospital, Tongji Medical College, Huazhong University of Science and Technology, Wuhan, China

**Keywords:** spinal cord injury, spinal cord repair, biomaterial scaffolds, cell transplantation, stem cell

## Abstract

Spinal cord injury (SCI) disrupts nerve pathways and affects sensory, motor, and autonomic function. There is currently no effective treatment for SCI. SCI occurs within three temporal periods: acute, subacute, and chronic. In each period there are different alterations in the cells, inflammatory factors, and signaling pathways within the spinal cord. Many biomaterials have been investigated in the treatment of SCI, including hydrogels and fiber scaffolds, and some progress has been made in the treatment of SCI using multiple materials. However, there are limitations when using individual biomaterials in SCI treatment, and these limitations can be significantly improved by combining treatments with stem cells. In order to better understand SCI and to investigate new strategies for its treatment, several combination therapies that include materials combined with cells, drugs, cytokines, etc. are summarized in the current review.

## 1 Introduction

SCIs are chronic and permanent injuries mainly caused by traffic accidents and unintentional falls that typically lead to compromised behavior, dysfunction, impairment, and a multitude of comorbidities (Wang et al., 2022). SCI is a major central nervous system (CNS) injury that represents a significant clinical challenge (Yuan et al., 2021). Currently, the global incidence of SCI is 27 million, with an annual addition of 93,800 new cases (GBD 2016 Neurology Collaborators, 2019). The estimated direct lifetime expenses per patient range from around $1.1 million to $4.8 million (Donovan and Kirshblum, 2018). The presence of SCI poses a significant economic and psychological strain on the affected individual, their family, and society.

SCI in humans are predominantly traumatic and can be categorized as complete or incomplete depending on the degree of trauma. Spinal cords undergo a complicated pathophysiological process after SCI. In an acute stage, the primary SCI initiates a multifaceted cascade of pathogenic changes, including hemorrhage, cellular damage, oxidative stress, axonal degeneration, and necrosis. Such changes lead to loss of neural tissues and over-production of inflammatory cytokines (Wu et al., 2022). In the following phases, host cells around the lesion respond to cytokines to remove cellular debris and initiate cellular proliferate that will confine the lesion. As a result, a fluid-filled cavitation is formed from the accumulation of different kinds of cells and extracellular matrix (ECM) after eradication of the injured tissue. Neurodegeneration persists despite attenuation of the inflammatory response during the chronic period. It should be noted that the remaining axons possess a restricted ability to regenerate and extend across the cavity that is surrounded by scar tissue (Anjum et al., 2020). The process of spontaneous recovery is strongly dependent on the presence of spared neural tissue and the activation of endogenous neural stem progenitor cells (NSPCs). The aforementioned repair methods fail to achieve a desirable level of functional recovery, particularly in patients with severe SCI (Liu et al., 2019).

Multiple strategies exist for the repair of SCIs, encompassing pharmaceutical interventions, bioactive molecules administration, cells and biomaterials transplantation, rehabilitation techniques, and electrical stimulation. Neurocompatible biomaterials exhibit considerable promise as either single or combined grafts for the therapeutic intervention of SCI. Biomaterials have the potential to fulfill various functions in the repair of SCI (Liu et al., 2019). These functions include serving as an ECM to occupy the lesion cavity and restore connectivity in the damaged spinal cord, transporting candidate cells or bioactive molecules, directing the regeneration of axons across the lesion site, attracting migration of endogenous NSPCs toward the lesion site, and impeding the formation of scar tissue (Zou et al., 2020). Recently, increasingly more studies are reporting the ability of functional biomaterials to regulate the spinal cord microenvironment, contributing to their high efficiency in repairing the spinal cord (Jin et al., 2019).

This study aims to comprehensively and systematically evaluate the pathophysiological alterations that occur following SCI. To investigate potential therapeutic targets for SCI, various biomaterials that are transplanted either individually or in combination with cells or biomolecules, with the goal of modifying the microenvironment in order to facilitate the repair of SCI, are discussed.

## 2 Pathophysiological process of SCI

SCI are classified within the field of pathophysiology into two categories: initial injuries and subsequent events. Besides the initial injuries, the determination of the severity of SCI is largely dependent on the secondary response (Eli et al., 2021), which can be classified into acute, subacute and chronic phases. The timeline for these phases is not strictly defined and may exhibit different manifestations in animal models and human patients (Adams and Gallo, 2018). For the purpose of this discussion, we will focus on the three periods that are commonly observed in human SCI.

### 2.1 Acute phase

**Figure 1:**
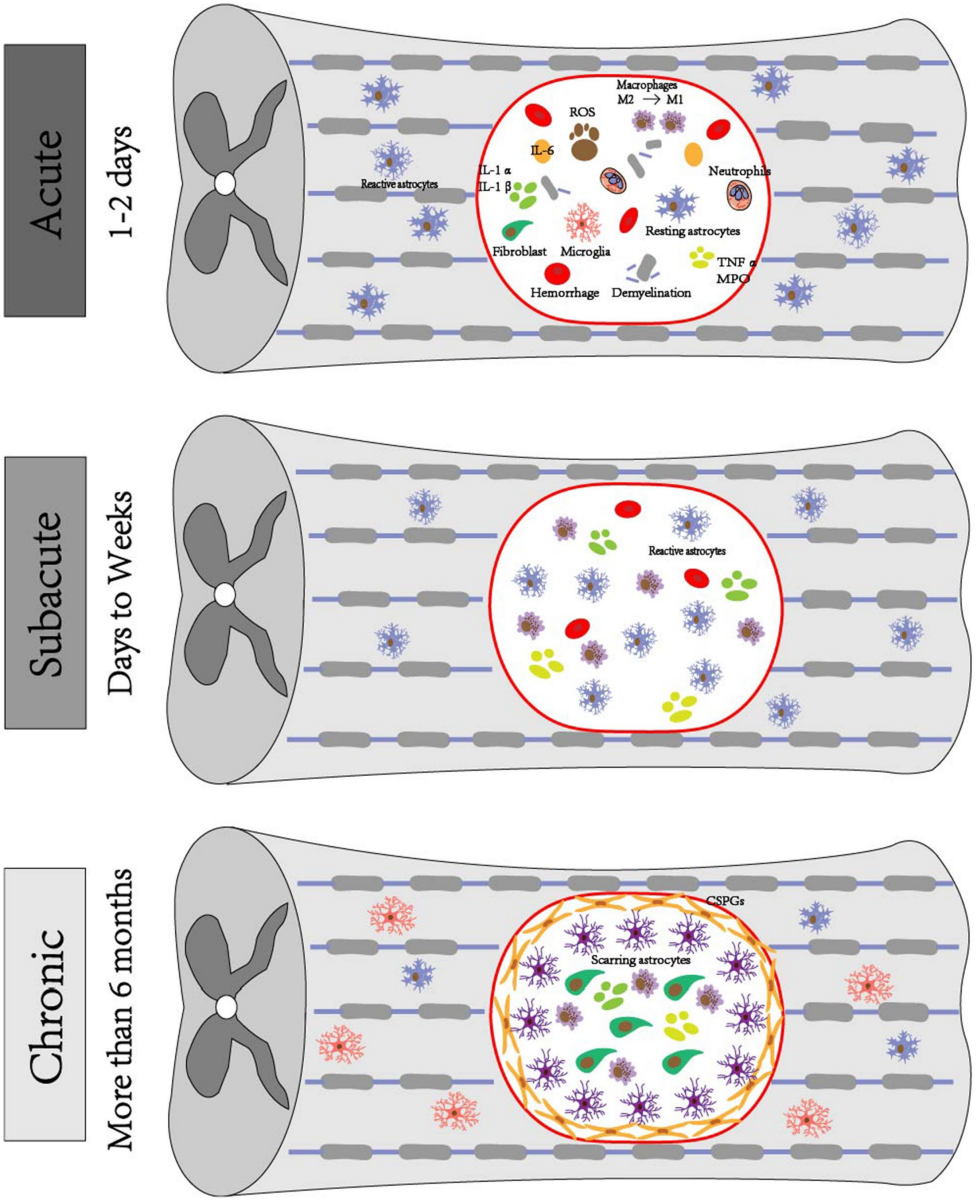
Pathophysiologic mechanisms of SCI. Acute phase: exhibits hematoma formation, Wallerian degeneration of distal axons with demyelination reaction, ROS production, inflammation occurs, ionic imbalance, macrophages phagocytose debris, astrocytes migrate toward the center of the lesion and encapsulate the damaged tissues, microglia are also activated and change their morphology, fibroblasts induce fibroblast reaction, and a large number of fibroblasts are deposited around the core of the lesion. Subacute phase: astrocytes form a glial scar and debris is further engulfed. Chronic phase: progressive generation of empty capsule cavities, myelin debris, deposition of GSPGs and scarred astrocytes.

The acute phase of SCI often manifests within 48 h following injury, resulting from spinal cord compression that is caused by fracture and dislocation of the spinal column (Zhou et al., 2022). After experiencing SCI, a pronounced inflammatory reaction occurs in the nerves, characterized by a significant influx of neutrophils into the affected region, within 2 h of the insult. At 24 h post-injury, there is a substantial accumulation of inflammatory cells at the location of the lesion. Neuronal toxicity is induced by the production of cytokines, free radicals, and other substances by activated neutrophils and macrophages (Gavriel et al., 2020). Both microglia and macrophages are capable of producing cytokines and growth factors. Additionally, macrophages have the ability to heal nerve injuries through the modulation of glutamate excitotoxicity and the promotion of axonal development and long-distance regeneration (Li et al., 2022). Shortly after sustaining an injury, there is a notable increase in pro-inflammatory cytokines in the spinal cord. The persistent increase in pro-inflammatory cytokines produced by neurons, such as tumor necrosis factor-α (TNF-α) and interleukin-1β (IL-1β), results in inflammation and an imbalance in cytokine release (Figure 1) (Zeng et al., 2019).

A variety of signaling pathways are also activated immediately after SCIs (Wang et al., 2021). Researchers have recently found that the Ras homolog family member A (RhoA) gene plays a large part in a number of pathological processes that occur following CNS injury and that involve the inflammatory response, glial scarring, and the presence of inhibitory factors. RhoA is a G-protein that associates with receptors located on the cellular membrane. Upon activation, it triggers the activation of the Rho-associated kinase (ROCK) signaling pathway (Roy et al., 2021). In the acute stage of SCI, activation of glial cells and macrophages results in the release of ECM proteins that impede the growth of axons. Notably, chondroitin sulfate proteoglycans (CSPGs) are among the ECM proteins secreted during this period. Some molecules, like CSPGs or Neurite outgrowth inhibitor-A (Nogo-A), bind to receptors on the surface of nerve axons to activate the RhoA-ROCK signaling pathway. This activation leads to phosphorylation of the light chains of myosin, resulting in the loss of motility of microtubule proteins (Stern et al., 2021).

Mitogen-activated protein kinase (MAPK) signaling pathway involvement is significant following SCI, as it facilitates the release of pro-inflammatory substances from microglia/macrophages (Liu et al., 2020). The MAPK family, which comprises extracellular signal-regulated kinase (ERK), p38 MAPK, and c-Jun N-terminal kinase, is a collection of signaling molecules that exert a significant influence on the development of pro-inflammatory cytokines (Pang et al., 2022). The activation of reactive oxygen species (ROS) triggers the MAPK signaling pathway, initiating an inflammatory response. Multiple studies have documented that activation of the MAPK signaling pathway induces inflammation by triggering downstream activation of the nuclear factor kappa-B (NF-κB) signaling pathway (Ramalingam et al., 2020). The NF-κB signaling pathway serves as a central regulator for a wide range of pro-inflammatory cytokines and is also activated by upstream ROS levels. In the context of the spinal cord, regulation of the NF-κB signaling pathway effectively hinders the expression of specific pro-inflammatory cytokines, namely IL-6, TNF-α, and IL-1β, by microglia (Liu et al., 2020). ROS have the ability to stimulate the polarization of M1 macrophages by activating the MAPK-NF-κB p65 signaling pathway. The ROS-MAPK-NF-κB p65 signaling pathway is a potentially targetable pathway for suppressing M1 macrophage polarization (Liu et al., 2022). Alginate is a natural polysaccharide extracted from the cell wall matrix of marine seaweeds and is often used in biological tissue engineering for scaffolding, cell encapsulation, and therapeutic drug release. Alginate also enhances toll-like receptor 4 (TLR4)-NF-κB-mediated phagocytosis in macrophages. Alginate enhanced phagocytosis in mouse macrophages by increasing TLR4 expression, activating the protein kinase B (AKT)/NF-κB and p8MAPK signaling pathways, and inducing macrophage activation (Tripathi et al., 2023).

### 2.2 Subacute phase

The subacute phase following SCI is a continuation of the acute phase of injury and includes the onset of lipid peroxidation, the inflammatory response, macrophage infiltration, axonal demyelination, and glial scar formation.

After the acute phase, the occurrence of ischemia and excitotoxicity results in disruption of intracellular and extracellular ion homeostasis. Specifically, dysregulation of calcium in neurons and glial cells leads to cell death. In animal models, it has been observed that elevated levels of intracellular calcium triggers activation of calpain, resulting in impaired mitochondrial functioning and cell apoptosis (Zivkovic et al., 2021). Moreover, the prolonged death of neurons and glial cells caused by ischemia, inflammation, and excitotoxicity results in the release of ATP, deoxyribonucleic acid (DNA), and potassium. These released molecules have the potential to activate microglia. Activated microglia, in association with other inflammatory cells, such as macrophages, polymorphonuclear cells and lymphocytes, enter the lesion, magnify the inflammatory response, and facilitate the ongoing death of neurons and oligodendrocytes (Quraishe et al., 2018). Astrocytes are also activated during this process and exhibit migratory behavior toward the central region of the lesion, where they subsequently envelop the compromised tissue as a protective measure against potential secondary injury to the adjacent healthy tissue (Lee et al., 2023). Oligodendrocytes (OLs) also play a role in subacute SCI, during which they are responsible for the production of myelin, a substance that envelops axons to create myelin sheaths. Additionally, oligodendrocyte progenitor cells (OPCs) move and multiply at the site of injury, eventually turning into myelinated OL (Figure 1) (Raffaele et al., 2021).

OLs are particularly sensitive to the toxicity of the acute SCI microenvironment induced by the secondary injury from SCI. The OLs envelop the axon and generate the myelin sheath by undergoing migration, proliferation, and differentiation (Raffaele et al., 2021). A significant portion of the myelin-producing cells that arise from demyelination caused by OL death cannot successfully regenerate, hence rendering other axons more susceptible to further damage (Kuhn et al., 2019). Following a decrease in OLs, OPCs undergo migration and proliferation at the site of the lesion. This process leads to OPC differentiation into myelinated OLs, subsequently rebuilding myelin around the demyelinated axons. This restorative process typically occurs around 2 weeks after the initial damage (Niu et al., 2021). As mentioned previously, activation of the RhoA-ROCK signaling pathway in microglia results in polarization of macrophages to the M1 type and the production of inflammatory factors (Zhang et al., 2022). Inhibition of myelin formation by Nogo-A has also been associated with activation of OLs and the RhoA-ROCK signaling pathway, and activation of RhoA inhibits differentiation of OPCs to OLs (Wang et al., 2021). Furthermore, cell migration and differentiation are blocked by CSPGs after activation of the RhoA-ROCK pathway in endogenous neural stem cells (Liu et al., 2022), and inhibition of RhoA activation in astrocytes reduces their proliferation and aggregation and extracellular matrix production (Stern et al., 2021).

A study suggests that co-transplantation of spinal cord derived neural progenitor cells (NPC) in conjugated polymer form with the Rho-kinase inhibitor fasudil can be used to treat SCI. By optimizing redox autolysis with a peptide carrier conjugate ligand with a short half-life in serum, they maximized the therapeutic potential of fasudil. In an *in vivo* model of acute and chronic SCI, polyglycolic acid (PGA) coupling promoted sustained and prolonged release of fasudil, enhancing neuroprotection and longevity. In addition, treatment of transplanted NPC with a poly-l-glutamate via a self – immolative redox-sensitive linker (PGA-SS-F) improved tissue contact and cell survival, suggesting that this may be an important tool for developing successful cell transplantation strategies (Giraldo et al., 2021).

Neurons and glial cells in the CNS, including the spinal cord, are particularly susceptible to oxidative and electrophilic stress due to their high polyunsaturated fatty acid content, high oxidative metabolic activity, and strong production of reactive oxygen metabolites and relatively low antioxidant capacity (Parida and Sahoo, 2023). Studies have shown that the presence of oxidative stress has the potential to exacerbate neuroinflammation and promote apoptotic processes. Therefore, oxidative stress is considered a marker of the secondary stage of SCI. Reducing oxidative stress may be an effective avenue for therapeutic intervention in SCI (Li et al., 2019). Oxidative stress plays an important role in the pathophysiology of SCI and is a marker of damage secondary to SCI in animal models (Ryan et al., 2024). Hence, it can be inferred that the activation of the antioxidant response holds promise as a therapeutic approach for addressing SCI (Zhang et al., 2023).

### 2.3 Chronic phase

When the inflammatory response subsides, the lesion moves into a chronic phase that is characterized by fibroglial scarring and cavitation, which are associated with myelin repair, vascular remodeling, changes in the ECM deposition, and reconnection of spared neuronal circuits. Fibroblasts migrate toward the vicinity of the lesion and proceed to substitute the ECM with fibrous connective tissue and deposits of collagen (Tran et al., 2018).

Astrocytes are the predominant population of glial cells inside the CNS. Following CNS damage, astrocytes undergo activation and transform into reactive astrocytes. These reactive astrocytes exhibit increased proliferation and induce morphological alterations, ultimately leading to the formation of glial scarring (Day and Milner, 2019; Garcia et al., 2023). However, this scar formation hinders axonal regeneration and impedes the process of spinal cord healing (Figure 1) (Zou et al., 2020). The defining characteristic of SCI is the development of cysts, which are closely linked to the TLR/myeloid differentiation primary response gene 88 (MyD88) signaling route in astrocytes. These astrocytes exhibit a persistent apoptotic response to necrosis via the TLR4/Myd88 signaling pathway (Li et al., 2022). The inhibitory effect on axonal regeneration during recovery is typically attributed to the presence of CSPGs that are generated by astrocytes in glial scarring. The initial stage following spinal cord ischemia–reperfusion injury primarily relies on TLR/MyD88 signaling and microglia activation. In later stages, microglia and astrocytes are responsible for TLR4/TIR-domain-containing adapter inducing interferon-β (TRIF) activation, which is accompanied by enhanced activation of MyD88 signaling. The TLR3 play a crucial role in TRF-dependent pathways, while the TLR4 possess the capability to activate TRF-dependent pathways subsequent to receiving signals from MyD88-dependent pathways (Hanafy and Jovin, 2024).

The primary factor influencing the development of glial scars is activation of the phosphatidylinositol 3-kinase (PI3K)/AKT signaling pathway. Blocking this pathway can diminish creation of glial scars. It has been demonstrated that blocking mammalian target of rapamycin (mTOR) activation decreases astrocyte proliferation, which decreases the development of glial scarring following SCI (Ryan et al., 2023). Axon regeneration following SCI is restricted by CSPGs, which are significant extracellular matrix constituents in glial scar tissues. CSPG expression patterns are regulated by phosphate and tension homology deleted on chromosome ten (PTEN) expression in the spinal cord (Yang et al., 2024). In addition, PTEN overexpression causes astrocytes to activate the PI3K/AKT/mTOR pathway, increases the expression of intermediate filament proteins (glial fibrillary acidic protein and vimentin), and reduces the expression of proliferating cell nuclear antigen (PCNA, a proliferation-promoting protein) (He et al., 2022). In SCI rats, PTEN overexpression also decreases the expression of CSPGs, inhibits the formation of glial scars, encourages the development of axons, and improves the recovery of nerve function. Thus, glial scar development is mediated by the PI3K/AKT/mTOR signaling pathway (Yang et al., 2024). A common and abundant extracellular matrix protein, fibronectin, is secreted as soluble dimers that form multimeric fibrils on the surface of cells. Transforming growth factor-β (TGF-β) is one of several cytokines involved in glial scar formation and CSPG expression. TGF-β induces the expression of CSPGs through activation of the PI3K/AKT pathway. Inhibition of TGF-β reduces glial scar formation after CNS injury (Chen et al., 2023). In general, activation of the PI3K/AKT signaling pathway promotes the proliferation of spinal cord astrocytes, whereas inhibition of this pathway reduces the formation of glial scars and promotes recovery after SCI (He et al., 2022).

Cell-adapted neural hydrogel (CaNeu) has been investigated as a carrier for adipose-derived stem cells (ADSCs); CaNeu hydrogel creates an anti-inflammatory microenvironment by inducing a polarity shift of recruited macrophages toward the M2 phenotype; ADSCs have an attenuated immunogenic response and a remarkable capacity for growth (Bacakova et al., 2018). ADSCs have been shown to promote healing of SCIs by secreting paracrine factors (Gao et al., 2019). ADSCs-mediated administration of CaNeu hydrogels has been shown to significantly inhibit neuroinflammation and apoptosis, a phenomenon involving the PI3K/Akt signaling pathway. These paracrine factors released by ADSCs activate the PI3K/AKT pathway, resulting in the phosphorylation of AKT and subsequent augmentation of M2 macrophages (Chang et al., 2023). Furthermore, upregulation of Bcl-3 and down-regulation of Bax impede apoptosis and contribute to the enhanced survival and proliferation of ADSCs within the microenvironment. Consequently, this process not only mitigates the inflammatory response following nerve damage but also facilitates the restoration of neural function. These results suggest that CaNeu hydrogels are a valuable transport vehicle for stem cell-based SCI therapy and offer a promising strategy for the treatment of CNS disorders (Yuan et al., 2021).

## 3 Application of biomaterial scaffolds in SCI

Scaffolds intended for SCI must satisfy a range of multifunctional prerequisites. The initial criterion pertains to biocompatibility, necessitating that the scaffolds be capable of being implanted within the body with little reactionary inflammation and rejection (Klabukov et al., 2023). Additionally, the scaffolds must be biodegradable as well as adaptable to the *in vivo* environment in order to facilitate optimal integration within the surrounding tissues. The scaffolds must also incorporate trophic factors, cells, agents, and other relevant components. Furthermore, the scaffolds should possess the ability to direct axon development and facilitate cell proliferation (Peng et al., 2023). Scaffold materials must offer an optimal milieu for the processes of cell adhesion, proliferation, and differentiation. Tissue engineering has witnessed extensive utilization of these entities, as evidenced by their widespread application in the field (Ding et al., 2021).

### 3.1 Hydrogel

Hydrogel is a type of biomaterial that has properties akin to those of gelatinous substances. Its primary purpose is to facilitate medication and cell transportation (Peng et al., 2023). Hydrogel exhibits favorable injectability and recoverability, rendering it suitable for administering chemicals to facilitate SCI healing. When treating SCI, hydrogel can be embedded within the spinal cord defects due to its structural similarity to soft tissue. It is also biocompatible and can be used as a scaffold to guide cell and axon growth (Wang et al., 2022).

#### 3.1.1 Natural hydrogel

The classification of natural hydrogels encompasses two main categories: protein hydrogels and polysaccharide gels. Protein hydrogels can be further classified into three main categories: collagen, gelatin, and matrix gel. Collagen is a protein found in the ECM of human tissues; hence, it is readily accessible for use within the protein hydrogel and exhibits limited exclusivity. Collagen offers binding sites that facilitate cell adhesion, proliferation, and differentiation (Yu et al., 2023). Hyaluronic acid itself plays an important role in rehabilitation of tissues following SCI, as it exhibits inhibitory effects on glial scar formation that are attributed to its ability to hinder lymphocyte migration and proliferation (Mahmoudi et al., 2024). Similarly, the combination of gelatin water gel with other cells or chemicals is commonly employed to create an antigenic environment that facilitates optimal conditions for nerve regeneration and proliferation (Zhang et al., 2021).

Alginic acid is classified as an anionic polysaccharide that has the ability to undergo crosslinking when exposed to multivalent cations. The utilization of alginic acid salt facilitates regeneration of nerve-injured cells (Wang and Sakiyama-Elbert, 2019). However, it is worth noting that alginic acid salt gel lacks a cell-binding domain. Consequently, alginic acid salt is commonly employed along with other reagents for the purpose of crosslinking. The alginate composition affects cell survival, with high levels of D-citrulline destabilizing the scaffold and high levels of L-glutamate inhibiting cellular metabolic activity in the absence of a coating. The establishment of axon bridges over lesions in the damaged spinal cord requires a growth substrate and guiding cues. When alginate hydrogels are used with capillary channels, the poly-L ornithine and laminin stably bind and improve cell adhesion and neurite growth by enhancing host cell infiltration into the injured spinal cord, in addition to enhancing axon growth *in vivo* (Schackel et al., 2019). Alginate has the advantage of low immunogenicity, long-term stability, and good biocompatibility *in vitro* and *in vivo*, allowing capillary hydrogels to serve as cell carriers. Its implantation at disrupted spinal cords promotes axonal regeneration (Huang et al., 2020). Furthermore, the combination of alginate hydrogels and Schwann cells (SCs) allows axon extension across the entire length of the channel without reducing the number of axons in the central portion of the scaffold. Brain derived neurotrophic factor (BDNF) has also been shown to promote axon growth through viral gene transfer to caudal tissue, however this growth is not sufficient to bridge SCI lesions. Overexpression of BDNF in the caudal end of the spinal cord promotes axon growth and penetration into the host parenchyma only when it is simultaneously injected with SCs into the caudal spinal cord (Hu et al., 2023). Alginate also enhances TLR-NF-kb-mediated phagocytosis of macrophages. Alginate activates the AKT/NF-kb and p38MAPK pathways by increasing TLR4 expression, which induces macrophage activation and enhances phagocytosis in mouse macrophages (Kim et al., 2023). While natural hydrogels have significant biocompatibility, their mechanical qualities are rather poor. When utilized in isolation, their limited biological activity renders them susceptible to local rejection responses. Thus, hydrogels are commonly employed in conjunction with bioactive compounds (Zhang et al., 2019).

#### 3.1.2 Synthetic hydrogel

Synthetic hydrogels are fabricated by the physical or chemical crosslinking of synthetic polymers. The manipulation of crosslinking chemistry in synthetic hydrogels allows for the modification of the compression modulus to align with the typical structure of the spinal cord (de la Vega et al., 2019). In general, there is a deficiency in terms of adhesion and activity among various materials, such as polycaprolactone (PCL), polyethylene glycol (PEG), polyvinyl alcohol (PVA), and poly(lactic-co-glycolic acid) (PLGA) and their derivatives (Peng et al., 2023). PEG is a biocompatible, hydrophilic synthetic hydrogel that has non-toxic properties. During the acute phase of SCI, the application of hydrogel impedes the inflammatory response and reduces oxidative stress, diminishing the formation of scar tissue and the capsule (Yari-Ilkhchi et al., 2024). PVA hydrogel has favorable elastic properties that mitigate the mechanical response exerted on the spinal cord. Additionally, it attenuates inflammation and minimizes the formation of scar tissue (Peng et al., 2023). Based on these characteristics, a new, easy-to-make hydrogel called agarose/gelatin/polypyrrole hydrogel (abbreviated as acid glycoprotein, AGP3) by varying the amounts of agarose and polypyrrole within the gel (Yang et al., 2022). *In vitro* culture experiments utilizing AGP3 show that it is biocompatible and supports neural stem cells (NSC) growth. AGP3 also promotes astrocyte cohesion, which stops activated astrocytes from infiltrating into the center of the lesion. Instead, the experiments showed that the glial cells migrated toward the lesion center, effectively suppressing astrocyte proliferation (Maiolo et al., 2021). Simultaneously, the AGP3 hydrogel restricted infiltration of monocytes and macrophages at the periphery of the lesion. Collectively, these properties of AGP3 confer the potential for it to improve the milieu surrounding the injury and decrease the activation of astrocytes *in vivo* (Song et al., 2020). Additionally, APG3 hydrogel impedes astrocyte activation due to its characteristics of elevated conductivity and reduced hardness (O'Shea et al., 2020). Consequently, this inhibitory action leads to a decrease in the expression of CSPGs, resulting in an efficient reduction in scar formation (Yang et al., 2022). Previous research conducted on living organisms has demonstrated that AGP3 hydrogel also provides cells with a milieu that is suitable for their biological processes. This microenvironment produced by APG3 hydrogel enhances the growth of nerves originating from within the organism and ultimately contributes to the restoration of normal bodily functions.

#### 3.1.3 Functional hydrogel

The utilization of functional hydrogel in conjunction with pharmaceuticals has the potential to enhance the efficacy of medications and, hence, augment their therapeutic outcomes. Functional hydrogels possess several key qualities making them suitable for SCI treatment, including a directed arrangement structure, conductivity, cell affinity, injectability, and multifunctionality (Peng et al., 2023). In a recent study, Yao et al. made a fibrin hydrogel (AFG) that was used as a cellular scaffold that contained stem cells and which was implanted into animal models. Within the model used, the AFG scaffold was able to direct the migration of nerve cells inside the host toward the AFG. Subsequently, the hydrogel material replaced the scaffold structure, resulting in formation of aligned cellular fibers and creating a physiologically active milieu that supports the regeneration of nerve cells *in vivo*. The AFG exhibited a superior capacity to facilitate axon orientation and growth due to its directed and aligned structure (Yao et al., 2018). Additionally, the high conductivity exhibited by hydrogel has the potential to facilitate restoration of the spinal cord functionality. And to impede the growth of astrocytes while concurrently facilitating cellular regeneration. Adhesion of hydrogels to the lesion typically requires association with integrin, which represents the predominant group of cell surface receptors at the spinal lesion. Involvement of the Arg-Gly-Asp (RGD) peptide of integrin in cellular adhesion processes throughout both developmental and reparative stages has been well established (Joung et al., 2020). Hydrogel injectability has also been demonstrated. For example, hydrogel can be administered via injection into affected areas through small incisions during surgical procedures, and hydrogels functionalized with lysine peptide can be utilized to fill the lesion cavity, reducing the inflammatory response and impeding the development of glial scar tissue (Liu et al., 2019).

### 3.2 Nanomaterial scaffolds

Nanomaterials with unique structures such as small size, large surface area and high specific surface area can be used as drug carriers to deliver therapeutic drugs to the target site, improve bioavailability and minimize the occurrence of side effects. In addition, nanoparticles can regulate axonal regeneration and restore signaling in the injured spinal cord (Wang et al., 2022). Due to their unique properties, nanoparticles can improve the mechanical properties of hydrogels, enhance surface interactions, and increase drug release rate and bioavailability. The addition of nanoparticles to hydrogels allows better access to tissues through capillaries and more effective delivery of therapeutic drugs to the site of injury (Li et al., 2023).

Numerous materials have been employed in clinical trials by leveraging the synergistic potential of nanotechnology and tissue engineering. The utilization of nanofiber scaffolds has emerged as a contemporary approach to promote axon development and directionality (Abbas et al., 2020). The nanofiber scaffold has a structural resemblance to the ECM, with a comparatively large surface area, cellular adhesion properties, and the ability to engage in cellular interactions. The electrospun nanofiber scaffold possesses a porous architecture and a substantial surface area, enabling the encapsulation of cells and pharmaceutical agents. The aforementioned attributes suggest that the nanofiber scaffold exhibits a high degree of suitability for the treatment of spinal cord damage (Cheng et al., 2021). The nanostructures included in regenerative scaffolds offer specific cues that effectively guide the growth and development of axons through adequate stimulation. Incorporation of multi-walled carbon nanotubes (MWCNTs) with exceptional physicochemical properties, along with biomimetic qualities, led to the development of three-dimensional carbon nanofibers (CNFs). Administration of chondroitinase ABC CNF has a substantial impact on the restoration of motor and sensory functions in animal models with long-term SCI through the facilitation of axonal growth. Utilization of MWCNTs within three-dimensional scaffolds offers an artificial exogenous milieu that directs and facilitates neural regeneration *in vivo* (Usmani et al., 2020). Several experimental findings have demonstrated that the nanofiber architecture of the nanofiber scaffold, along with its implantation, significantly diminishes the infiltration of inflammatory cells and the development of astrocyte scars at lesion sites. Moreover, integration of the scaffold with neurotrophic-3 (NT-3) offers an additional preventive measure against scar formation (Sun et al., 2020).

### 3.3 Fiber material scaffolds

Electrospinning produces nanostructured polymer fiber materials, also known as electrostatically spun fiber materials (EFM). These electrospun fibrous materials can be used in many biomedical fields such as wound dressings, tissue engineering and medical textile materials. After decades of research, electrospun nanofibers have been widely used for drug delivery in porous dispersion membranes, solid dispersions, and various controlled-release dosage forms due to their high surface-to-volume ratio, high porosity, ease of fabrication, and adaptability (Cheng et al., 2018). Electrospun nanofiber scaffolds can also mimic the extracellular microenvironment. Due to their large specific surface area, one-dimensional longitudinal properties and wide range of biological functions, this property allows them to be used for tissue regeneration, especially for cell adhesion processes (Dinuwan Gunawardhana et al., 2024). The controlled release of electrospun filament materials can be used in the pharmaceutical field and biomedical applications. Drugs can be loaded into electrospun filament materials and the release of drugs from the nanofibers can be regulated by adjusting the process and formulation parameters. Drug release from the fibers can be attributed to dissolution, absorption, subsequent diffusion through water-filled pores, and controlled degradation of the polymer (Hu et al., 2024). Hatamzadeh et al. reported a novel conductive nanofiber scaffold that can be used for tissue engineering requiring electrical activity, including nerve, bone, and muscle reconstruction (Massoumi et al., 2019).

### 3.4 Three-dimensional material supports

Three-dimensional bioprinting technology allows the size and shape of the support to be adapted to the needs of the wound. Many biomaterials have been used to design three-dimensional supports for SCI repair. It was found that in a rat model of SCI, a novel biocompatible biocomposite consisting of functional chitosan, hyaluronic acid derivatives, and matrix gel rapidly gels, spontaneously establishes covalent cross-links, maintains the viability of NPCs, and efficiently promotes axonal regeneration (Liu et al., 2021). It has been found that 3D printed supports deliver uniform mesenchymal stem cells (MSCs) and SCs from bone marrow in a specific spatial arrangement, promoting the formation of intercellular junctions and the differentiation of specific cells (Wang et al., 2021). The three-dimensional cryoconstruction technique can maintain the biological activity of the delivered cytokines. Collagen/chitosan prepared using three-dimensional frozen construct technology integrated brain-derived neurotrophic factors, filled the injury gap, promoted the regeneration of nerve fibers, facilitated the formation of synaptic connections, and promoted the regeneration of myelin sheaths at the injury site of SCI rats (Liu et al., 2021).

Joung et al. fabricated physiologically active neuronal networks by pinpointing clusters of NPCs in the spine during assembly and controlling the position of the clusters using a dot-printing method (Joung et al., 2018). Koffler et al. utilized microscale printing to fabricate complex neural network structures and three-dimensional bionic hydrogel scaffolds filled with NPCs, which facilitated axon regeneration in SCI mice and stimulated synapses at the top and bottom of the implant connect with the NPCs in the scaffold, significantly improving the functional prognosis. In addition, the scaffold can be expanded according to the size of the human spinal cord and the geometry of the lesion (Suzuki et al., 2023). In short, by adjusting the shape and size of the scaffold, the damaged area can be well filled (Feng et al., 2023).

## 4 Combined application of biomaterials

Simonetta Papa et al. focuses on the therapeutic effects of multiple cells such as MSCs, SCs, embryonic stem cells (ESCs), etc. and multiple materials such as hydrogels, nanofibers, etc. on SCI. Unlike this article, this paper focuses on the clinical treatment and repair of SCI by combining multiple materials with multiple cells, growth factors, and drugs, and explores how to treat SCI from more mechanisms (Papa et al., 2020).

Achieving desired outcomes in SCI healing is seldom attained with the utilization of cell regeneration or materials in isolation. Hence, diverse strategies involving the integration of cells, materials, drugs, ions, and other factors are employed to facilitate proliferation of stem cells or SCs by leveraging their synergistic effects (Ji et al., 2023). A combined approach aims to establish a microenvironment conducive to the sustained survival and replication of specific cells involved in SCI healing over an extended period of time. Subsequently, differentiated cells can effectively engage in the restorative processes of the spinal cord (Cerqueira et al., 2018).

### 4.1 Biomaterials combined with cells

#### 4.1.1 Cationic cross-linked hydrogels and stem cells synergize to regulate the microenvironment

Pluripotent stem cells are undifferentiated and self-renewing cells that include all types of stem cells found in adult organisms, such as bone marrow MSCs, hematopoietic stem cells (HSCs) or NSCs (Mirzaei et al., 2018). Bone marrow MSCs give rise to adipocytes, osteoblasts and chondrocytes, while hematopoietic stem cells can differentiate into all cell types of the hematopoietic system. However, studies have shown that adult stem cells can also form cells of other cell lines based on molecular signals from the transplantation microenvironment (Paliwal et al., 2021). This phenomenon, known as stem cell plasticity, greatly expands the potential use of stem cells in the treatment of a wide range of diseases, including SCI.

**Figure 2:**
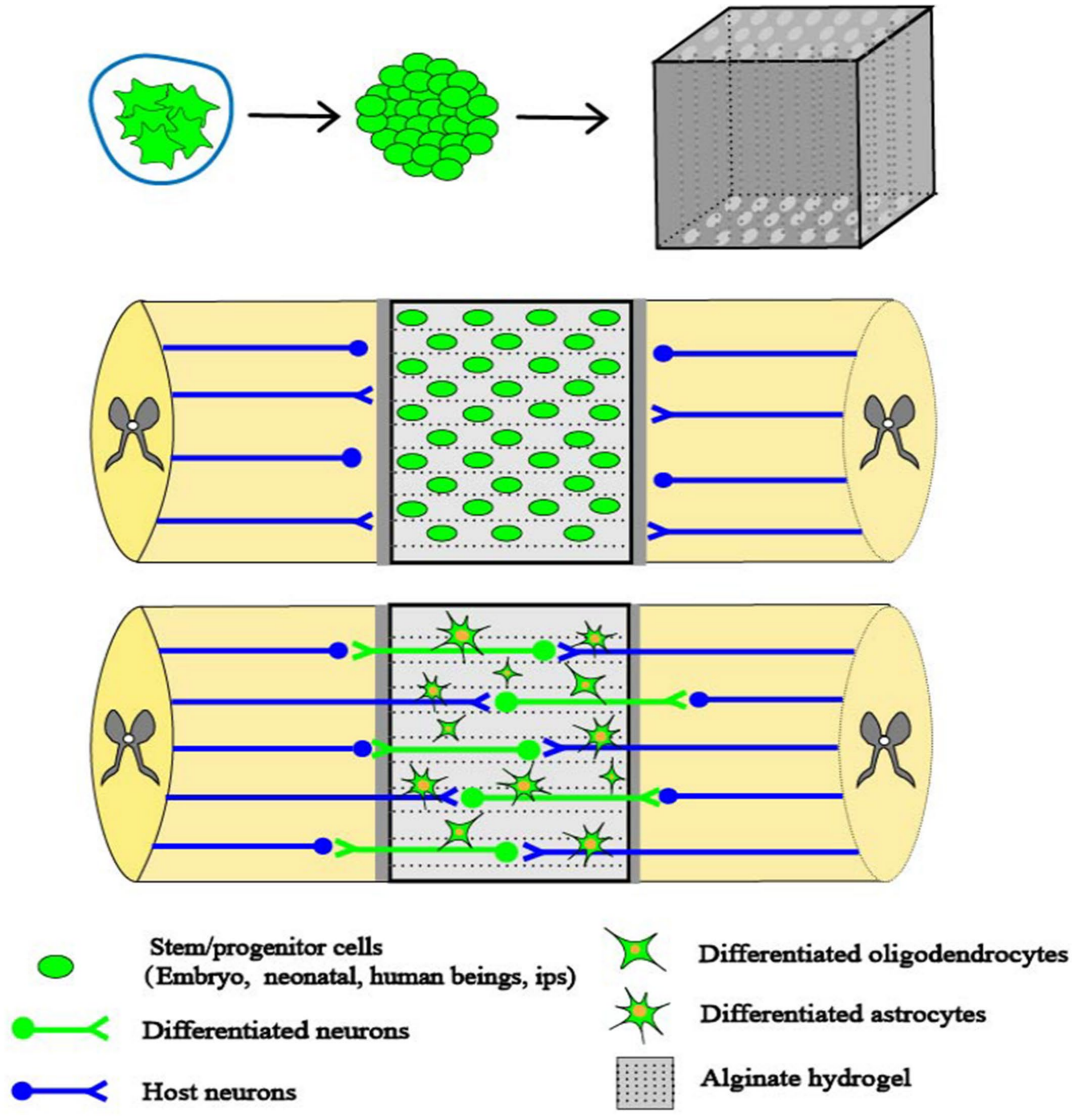
Combinations of cells and capillary scaffolding. SCI creates cavities that impede axonal signaling. Alginate capillary hydrogels have well-aligned channels that propel cell growth in specific directions. When stem cells derived from embryonic cells are implanted into the alginate hydrogel, they occupy the voids and promote axonal regeneration. Stem cells are able to establish synapse-like connections between host axons and neurons formed by the stem cells within the alginate hydrogel channels, thus promoting spinal cord regeneration (Szymoniuk et al., 2022).

Currently, pluripotent stem cells, such as MSCs and spinal cord NSCs, have been extensively investigated as potential therapeutic agents for the treatment of SCI in clinical settings (Figure 2) (Szymoniuk et al., 2022). MSCs are pluripotent stem cells and have the greatest potential of all stem cell types to treat SCI (Attia and Mashal, 2021). MSCs are easy to isolate, proliferate rapidly, and can be obtained from patients (Peng et al., 2019). Clinically used MSCs can be derived from autologous sources such as bone marrow and adipose tissue. In addition, bone marrow MSCs can be derived from allogeneic sources such as umbilical cord blood, placenta and amniotic fluid (Yang et al., 2024). Bone marrow MSCs are characterized by low immunogenicity and do not elicit the strongest immune response of the above sources (Muthu et al., 2021).

Spinal cord MSCs transplanted into damaged nerves increase the number of neurons capable of generating electrical impulses and facilitate the re-establishment of broken axonal connections (Kumamaru et al., 2018). Hence, stem cell transplantation has been investigated as a therapeutic approach for enhancing axonal regeneration, as documented in previous studies (Kim et al., 2021). HSCs, as pluripotent stem cells, can differentiate into various types of blood cells and lymphocyte lineages. HSCs transplanted into the SCI microenvironment exert their therapeutic activity through differentiation and release of a large number of cytokines and neurotrophic factors. The differentiation capacity of hematopoietic stem cells in the SCI microenvironment includes conversion to astrocytes, neuroprotective glial cells and OLs (Upadhyayula et al., 2021). The synergistic effects and regenerative potential exhibited by compounds and small molecules, in conjunction with the presence of endogenous NSCs, may facilitate the production of new neurons to mend the spinal cord and reinstate its functionality. This development presents a novel and auspicious avenue for the therapeutic intervention of SCI.

Alginate is a negatively charged polysaccharide derived from seaweed. As a negatively charged polysaccharide, alginate poses a restriction on the attachment and viability of various cells. To overcome this constraint, the introduction of cations facilitates formation of hydrogels through the establishment of ionic bonds (Valenti and Vacca, 2023). Various divalent cations have been investigated in the context of tissue engineering when combined with alginate hydrogels. Diffusion of alginate divalent cations within the alginate solution has been observed any may result in increased stability of the hydrogel scaffolds. Among these cations, calcium (Ca^2+^) is commonly utilized, particularly in its ionic form (Yang et al., 2019). The presence of Ca^2+^ solution facilitates the crosslinking of alginate, leading to the formation of alginate hydrogels (AHs). These AHs have the ability to develop capillary structures. To address SCI, capillary structures were used to transplant stem cells and introduce AHs into the capsule lumen at the site of injury (Slovinska and Harvanova, 2023). Stem cells have the ability to undergo differentiation, and through their growth and proliferation inside the capillary structure they may potentially alleviate the obstruction of tissues at the site of damage. By establishing a connection between a specific region of the injured site and the differentiated cells, it may be possible to relieve the effects of SCI. In addition, the capillaries create a space where NSPCs facilitate axonal proliferation that originates from the host. These capillaries establish connections with neurons produced from NSPCs, resulting in synapse-like contacts. This interaction has the potential to enhance electrophysiological conduction and boost functional recovery at the site of injury (Figure 2) (Liu et al., 2021).

#### 4.1.2 Hydrogels and stem cells synergize to regulate the reactive oxygen microenvironment

The presence of a peroxidized environment is a significant contributor to secondary injury and impedes the recovery of neural tissue. MnO_2_ has an antioxidant effect, and NPs dot hydrogel has an antioxidant and cell protection effect that alters the pathological microenvironment, to providing a good therapeutic prospect for neural tissue repair (He et al., 2023). In the experimental setting, when MSCs and MnO_2_ NPs were introduced into a multifuctional hydrogel, the resulting microenvironment facilitated the adherence and viability of MSCs, as well as the establishment of connections between neural tissues. The presence of MnO_2_ NPs within the hydrogel thus exerted a significant influence on the recovery of neural tissues and the regeneration of MSCs. This effect was attributed to the regenerative properties of nerves and the synergistic interaction between MSCs and MnO_2_ NPs. This may be due to the fact that the MnO_2_ NPs in the hydrogel created a less oxidative environment, which greatly improved the ability of MSCs to live and regenerate. At the same time, the investigators also found that brain tissue regrowth and functional recovery was significantly limited when MSCs were not present in the hydrogel. Thus, using hydrogel with dispersed MnO_2_ NPs may speed up spinal cord regeneration and augment the recovery of normal spinal cord function. This is achieved through the regulation of the reactive oxygen microenvironment and the synergistic effects of MSCs (Figure 2) (Li et al., 2019).

Cerium oxide nanoparticles (CeNPs) have properties that mimic those of albumin, and their dispersion into methacryloyl gelatin (CelMA) leads to the formation of a hydrogel with the ability to scavenge ROS. This scavenging process enhances functional restoration after SCI by impeding the development of neuroglial scars and facilitating the growth of neurons at the lesion. CelMA-Gel can enhance the survival and differentiation of NSCs and promote nerve fiber regeneration by alleviating oxidative stress. Hence, the utilization of a ROS-scavenging hydrogel in conjunction with NSC therapy presents a very encouraging therapeutic approach for addressing SCI (Liu et al., 2023).

#### 4.1.3 Biomaterials and stem cells synergize to alleviate the neuroinflammatory microenvironment

Both biomaterial scaffolds and MSC transplantation have been shown to be effective as treatments for SCI when used individually. The use of biomaterial scaffolds and MSC transplantation in conjunction has been shown to have synergistic effects. In contrast to monotherapy, utilization of combination therapy attenuates apoptosis and inflammation in nerve cells. Furthermore, combination therapy facilitates the formation of myelin in nerve fibers and augments synaptic connectivity (Giorgi et al., 2024). Notably, the reduction in astrocyte formation and the secretion of CSPGs by glial cells plays a significant role in mitigating fibrosis during the acute phase of SCI and in the subsequent formation of glial scars (Metwally et al., 2023).

Adipose-derived mesenchymal stem cells (ADMSCs), isolated from subcutaneous fat by liposuction, are minimally traumatized, easier to extract, and expand faster. Their ability to differentiate in multiple directions and to secrete a variety of bioactive factors make them promising for a wide range of applications in autologous stem cell transplantation. In addition, the potential of ADMSCs in neural differentiation has been recognized (Litak et al., 2022), and these ADMSCs-derived neural cells have been shown to promote neural repair. More importantly, ADMSCs exhibit low immunogenicity and tumorigenicity.

A thermosensitive hydrogel made of quaternary chitosan and glycerophosphate (HACC/GP) has also been used to treat SCI, along with adenovirus and MSCs that carried the neurotrophic factor gene for glial cell-derived neurotrophic factor (GDNF) (Ad-rGDNF) (Tsung et al., 2023). The survival and growth of MSCs can be supported by human astrocyte-conditioned medium (HACC) within the scaffold material. Previous studies have found that HACC hydrogel scaffolds have significant slow-release properties for target genes and proteins. In addition, based on the property of permanent positive charge, they found that HACC could protect target genes from degradation by intracellular proteins and DNA degrading enzymes. Combined with the findings, it can be demonstrated that HACC hydrogel scaffolds also have the advantage of promoting GDNF secretion from stem cells. In addition, previous studies have also shown that GDNF significantly improved the viability of bone marrow mesenchymal stem cells (BMSCs) during induction and promoted the proliferation of BMSCs (Yuan et al., 2024). The results of the study showed that after HACC/BMSCs were administered, the expression levels of NF200 and glial fibrillary acidic protein (GFAP) were significantly higher in the area of the spinal cord lesion. GFAP is glial fibrillary acidic protein that acts as an inflammatory protein and that is found mainly in astrocytes. Consequently, GFAP is commonly employed as a reliable indicator of astrocyte activation. In short, adding bone marrow-derived MSCs to hydrogels made of HACC/GP may help neural cells grow and become more specialized. Furthermore, this approach has had a positive impact on the restoration of neurological function following spinal cord damage. Thus, one possible way to induce neural differentiation is to use MSCs implanted in hydrogels made of HACC and GP, along with the adenovirus-mediated delivery of growth factors. This technique has promise as a therapeutic mechanism for addressing forthcoming neurological disorders (Huang et al., 2021).

Peptides can be employed to modify AHs, facilitating the binding of L-ornithine and laminin to the alginate hydrogel matrix. This modification enables enhanced infiltration of neuronal cells, thereby promoting increased cell adsorption and axonal development. Transplantation of astrocytes into the hydrogel results in enhanced survival of these cells, which in turn promotes a higher axonal development and facilitates the connection and integration of short-distance axons at the site of injury. Prior research has demonstrated that the viability and proliferation of bone marrow MSCs and chevron cells within unencapsulated channels in the presence of AHs (Zhou et al., 2023). *In vitro* investigations have demonstrated that astrocytes exhibit a greater affinity for residing within channels that are enclosed within AHs. This arrangement facilitates the establishment of a distinct demarcation between the host cells and the grafted cells, consisting of bone marrow MSCs and SCs. SCs are a type of glial cell that is responsible for myelination in the peripheral nervous system. Following a SCI, the injured region is infiltrated by SCs, which facilitate the process of tissue healing and the synthesis of myelin. Spinal cord damage is commonly characterized as an injury to the dorsal root entry zone of the spinal cord. It has been shown that SCs have the ability to migrate a significant distance both cranially and caudally from the location of the lesion. This migration of SCs facilitates the regeneration of axons and the production of myelin sheaths (Cerqueira et al., 2018). SCs have undergone a thorough investigation in which it has been determined that they possess neuroprotective properties. Thus, SC culture and transplantation has been employed as a therapeutic approach to addressing SCI. Conversely, astrocytes promote enhanced integration between the host cells and the transplanted cells. The promotion of cell attachment, migration, and axon growth is facilitated by the laminin-binding properties of the alginate hydrogel, which augments cellular infiltration. Additionally, the presence of the astrocytic matrix within the scaffold ensures a continuous connection between the host and the alginate hydrogel channel, hence further enhancing axon growth and facilitating nerve repair (Figure 2) (Schackel et al., 2019).

Šárka Kubinová describes the repair of SCI by combining synthetic hydrogels and biohydrogels with a variety of cells, and the article Magnetic Stem Cell Targeting is a minimally invasive solution that enhances the retention of transplanted cells at the site of injury and thus improves therapeutic efficacy, an effect that can manipulate the spatial organization of the stem cells and create an interconnected cellular network that opens new perspectives in tissue engineering. Compatible magnetic nanoparticles and a detailed understanding of the interactions between magnetic nanoparticles and biological systems are fundamental for the long-term clinical viability of this technology (Kubinova, 2020) (Table 1).

**Table 1:** Stem Cells biomaterial SCI studies.

SCI model	Biomaterial	Cells types	Outcome	Reference
SD rat T8 transection	Ca^2+^ and alginate cross-linking	NSPCs from SD pregnant rats embryos expressing green fluorescent protein	AH scaffolds consisting of Ca2+ bound to NSPC promote NSPC survival and differentiation and support linear regeneration of short axons	Zhou et al. (2022)
F344 rat C5 transection	Alginate hydrogel coated PLO/laminin	Adult rat astrocytes	Improvement of cell attachment, cell migration and axon growth by PLO and laminin coating of alginate hydrogels	Schackel et al. (2019)
SD rat T10 transection	MnO2 NPs dot hydrogels	MSCs derived from amniotic membrane of human placenta	MSCs encapsulated MnO2 np dots hydrogel contributes to neurological recovery by inhibiting glial scarring and promoting nerve fiber regeneration in lesions	Li et al. (2019)
SD rat T9–T10 transection	CaNeu hydrogel	ADSCs derived from rat epididymal adipose tissue	Paracrine factors secreted by ADSCs enhance M2 polarization of recruited macrophages, significantly reducing neuroinflammation at the site of injury.	Yuan et al. (2021)
SD rat T8–T9 transection	Porous thermosensitive HACC/β-GP hydrogel	BMSCs derived from rat femur	HACC/β-GP hydrogel scaffolds have significant benefits on neural differentiation of BMSCs and promote recovery of neurological function after spinal cord injury in rats	Huang et al. (2021)
F344 rat C5 transection	Alginate hydrogels	Adult rat SCs	Alginate hydrogels with anisotropic capillaries supported the survival of transplanted SCs and guided supraspinal and native spinal cord downstream axons through and over the lesion site.	Huang et al. (2020)
SD rat T7–T9 transection	Immobilizing N-cadherin on linearly ordered collagen scaffold (LOCS)	NSPCs derived from the telencephalon of neonatal rats	LOCS-Ncad promoted the differentiation of NSPCs into neurons and regeneration of axons, and inhibited the deposition of CSPGs.	Liu et al. (2020)

### 4.2 Materials combined with medicine

Assembly using organic polymeric material I-5 hydrogel in a solution mixed with human recombinant arylsulfatase B (ARSB) enables the effective transportation of diverse hydrophobic polytubes and pharmaceutical substances to facilitate the extended release of medications. *In vivo* experiments were performed using adult female Sprague–Dawley (SD) rats, the contusion injury model that has the greatest correlation with human traumatic spinal cord injury. 10 μL of I-5/ ARSB complex containing 1.9 μg of human recombinant ARSB was dissolved in 10 wt % I-5 polymer micellar solution. For the I-5 only group, the 20% I-5 polymer micelle solution was mixed with an equal amount of PBS, and 10 μL of the final 10% I-5 solution was injected (Pranantyo et al., 2024). Consequently, organic polymeric material I-5 hydrogel solutions containing ARSB exhibit a prolonged therapeutic impact (Seo et al., 2019). Following CNS injury, CSPG production is upregulated and inhibits axonal regeneration at the location of the lesion. CSPGs are synthesized by several cell types, including astrocytes, fibroblasts, and macrophages, and are known to significantly influence fibrosis and wound healing. CSPGs serve as a substrate that facilitates fibroblast aggregation, potentially leading to increased fibrosis and scarring (Wu et al., 2022). Currently, the degradation of CSPGs involves the use of ARSB, which selectively inhibits the presence of 4-CS sulfate (C4S) glycosaminoglycans (GAGs) in the sugar chain of CSPGs (Pearson et al., 2018). The dominance of C4S in the sugar chain hinders axon regeneration following injury. Therefore, the application of ARSB can enhance the regenerative potential of axons by creating a more favorable ECM environment within the hydrogel. I-5 hydrogel solutions containing ARSB has the potential to mitigate the fibrotic milieu of the ECM caused by the imidazole component of the I-5 hydrogel. Additionally, it has been observed that this hydrogel simultaneously enhances axon regeneration while inhibiting fibrogenesis. By making it easier for axons to connect between tissues, the newly made ECM helps to repair and regenerate the spinal cord. Importantly, the altered microenvironment of the ECM due to the use of ARSB does not prevent the I-5 hydrogel from performing its role in preventing tissue defects (Zhao et al., 2018).

Betulinic acid (BA) is a natural pentacyclic triterpenoid that is extensively utilized in Chinese herbal medicine. Importantly, BA has the ability to cross the blood–brain barrier, which is important in the treatment of CNS lesions. C57BL/6 mice were used for the experiments, and all drugs were dissolved in 2% DMSO saline and administered intraperitoneally using a standard experimental cage with a light/dark cycle for 12 h. All mice were individually housed. Mice were randomly divided into 5 groups:BA group was injected with 20 mg/kg BA daily after SCI. BA group was injected with 20 mg/kg BA daily after spinal cord injury. BA+ 3-methyladenine (3MA) group and BA+ CC group were injected with 3MA (15 mg/kg) and dorsomorphin (Compound C, 1.5 mg/kg) 30 min prior to the administration of BA (20 mg/kg). BA+3MA group and BA+ Compound C (CC) group were injected with dorsomorphin (Compound C, 1.5 mg/kg) 30 min prior to the administration of BA (20 mg/kg). The dose and time of BA administration were based on previous studies (Li et al., 2019). Previous studies have shown that BA exerts neuroprotective effects by promoting autophagy, reducing ROS, and inhibiting inflammation. BA activates the AMPK-mTOR-TFEB (transcription factor EB) signaling pathway, which enhances autophagy in SCI. Increased autophagy induces mitogenic autophagy and reduces ROS accumulation, thereby inhibiting focal death. Ultimately, these effects of BA improve prognosis after SCI (Wu et al., 2021).

Kaempferol is a well-characterized natural polyphenolic, which has antioxidant and anti-inflammatory properties and is protective against a variety of neurological disorders. Adult male SD rats of 8 weeks of age were used for the experiment. Rats were randomly divided into 5 groups (6 rats per group): Sham group, SCI group, SCI + kaempferol (25 mg/kg) treatment group, SCI + kaempferol (50 mg/kg) treatment group, SCI + kaempferol (100 mg/kg) treatment group. The SCI + kaempferol (25 mg/kg,50 mg/kg, 100 mg/ kg) treatment group were pretreated with kaempferol (suspended in 0.5% CMC-Na) at dose of 25 mg/kg,50 mg/kg, 100 mg/kg once daily for 7 days before operation and 3 days after operation by i.g. The Sham group and SCI group were treated with equal volume of 0.5% CMC-Na solution by i.g. (Liu et al., 2021). Kaempferol pretreatment of BV2 cells reduces ROS production through inhibition ofnNicotinamide adenine dinucleotide phosphate (NADPH) oxidase 4, which in turn inhibits phosphorylation of p38 MAPK and JNK. As a result, NF-κB p65 nuclear translocation is prevented and pro-inflammatory transcription is repressed (Yu et al., 2019). Kaempferol also inhibits the focal death-associated protein and reduces the release of IL-18 and IL-1β. Collectivley, these data show that kaempferol reduces oxidative stress and inflammatory responses by down-regulating ROS-dependent MAPKs-NF-κB and pyroptosis signaling pathways (Li et al., 2019).

### 4.3 Biomaterials combined with cytokines

Polypropylene fumarate (PPF) exhibits notable mechanical strength and offers a robust environment for tissue repair. Moreover, the parallel arrangement structure of PPF scaffolds facilitates the guidance of nerve tissue regeneration and enhances the connectivity of nerve axons (Chen et al., 2018). The integration of platelet-rich fibrin (PFF) with collagen-based biomaterials results in the development of a novel polymer delivery method that exhibits biocompatibility. The system encompasses a structural component known as the collagen-binding domain-NT3 (CBD-NT3) of neurotrophic factor-3. The linear collagen scaffolds exhibit minimal antigenicity and favorable biocompatibility. Furthermore, the incorporation of neurotrophic factors facilitates the *in vitro* proliferation of nerve axons and the regeneration of nerve fibers (Han et al., 2019). Collagen treatment that incorporates PPF and CBD-NT3 has been shown to enhance the microenvironment, decrease the occurrence of glial and fibrotic scarring in lesions, and facilitate neuronal survival and axonal regeneration following SCI. This treatment not only provides a conducive microenvironment for neuronal regeneration but also enhances the connectivity of neural tissues and strengthens the functionality of the spinal cord, thereby improving the overall repair process following SCI. This combination of biomaterials and growth factors has great potential for future research and clinical treatments (Chen et al., 2018).

Vascular endothelial growth factor (VEGF) has the ability to promote the migration and proliferation of vascular endothelial cells (Ju et al., 2019). However, the biological half-life of VEGF is relatively short, so controlling the rate of its release is a key aspect of the treatment of SCI. A functional composite scaffold composed of heparin, collagen, and VEGF in which the composite scaffold has good mechanical properties and is able to control the rate of VEGF release may be a viable treatment for SCI. According to the study conducted by M. Dzietko et al., it was shown that VEGF has a dual role in facilitating angiogenesis and safeguarding neurons following brain damage (Labusek et al., 2023). Hence, through the incorporation of various growth factors into the material scaffolds, these scaffolds serve the dual purpose of immobilizing and preserving the activity of the growth factors. Additionally, by regulating the rate at which the growth factors are released, these scaffolds can effectively stimulate axonal growth, facilitate stem cell differentiation, and facilitate tissue regeneration. Consequently, this approach holds significant promise as a therapeutic strategy for addressing brain injuries.

A hydrogel composed of PCL supplemented with fibroblast growth factor 2 (FGF2), epidermal growth factor (EGF), and GDNF has the capability of conferring structural reinforcement to tissues while concurrently facilitating the adhesion and proliferation of neural cells (Chin et al., 2023). FGF2 and EGF have been shown to facilitate the process of axonal regeneration. Additionally, glial-derived GDNF has been observed to lead regenerating axons toward its source. Researchers conducted an experiment in which they implanted a composite scaffold consisting of PCL and cytokines into the site of SCI in rats and showed that the composite scaffold effectively directed the growth of neuronal cells and facilitated axonal regeneration. Furthermore, the composite scaffold created a favorable microenvironment that supported the survival and proliferation of cells. This innovative composite scaffold is regarded as a viable therapeutic approach for the treatment of spinal cord damage (Wang et al., 2020).

The potential of stem cells in the therapeutic management of SCI is highly promising. However, the rates of survival and differentiation of stem cells are currently limited. Notably, the regulatory functions of stromal cell-derived factor-1 (SDF-1) and its receptor chemokine receptors C-X-C chemokine receptor type 4 (CXCR4) are crucial in governing the survival and differentiation processes of stem cells (Janssens et al., 2018). The SDF-1/CXCR4 signaling pathway is implicated in the modulation of cellular developmental and migratory activities (Qing et al., 2023). Additionally, it plays a significant role in the facilitation of nerve regeneration and repair mechanisms. These findings indicate that the interaction between SDF-1 and CXCR4 enhances the viability and specialization of BMSCs, as well as facilitates their movement toward affected areas in a laboratory setting. These effects are achieved by activating the focal adhesion kinase (FAK)/PI3K/AKT pathway. When sodium alginate (SA)/collagen type I (CoI)/SDF-1 hydrogel is implanted into bone marrow MSCs, it facilitates the stable release of SDF-1. This controlled release of SDF-1 creates an optimal microenvironment that supports the viability and differentiation of BMSCs. It has been shown that applying BMSCs/SA/Col/SDF-1 hydrogel can heal nerve injuries and reduce the swelling, inflammation, and oxidative stress in the brain that occurs following nerve injuries. BMSCs/SA/Col/SDF-1 hydrogel also attenuated oxidative stress, and the implantation of hydrogel significantly inhibited the apoptosis of neuronal cells after SCI (Guan et al., 2019).

### 4.4 Biomaterials combined with other strategies

#### 4.4.1 Biomaterials combine with miRNAs to construct microenvironments suitable for axonal regeneration

miRNAs are a class of small, non-coding RNAs that play a crucial role in the regulation of genes and proteins involved in maintaining the integrity of the CNS. These miRNAs have significant roles in various processes, such as neural development, functional circuit formation, axonal regeneration at injury sites, and sensory axon regeneration (Ionescu et al., 2023). Furthermore, the concurrent administration of axonal miRNAs and methylprednisolone reduces inflammation and augments expression of those genes related to ECM deposition. Additionally, methylprednisolone increases levels of miRNAs inside axons, therefore facilitating the regeneration of myelin and enhancing functional recovery. The utilization of miRNAs in conjunction with methylprednisolone to modulate the process of axonal regeneration is a viable and efficacious strategy for addressing spinal cord damage. In a previous study, rats with spinal cord transection were subjected to transplantation of 3D fiber hydrogel and axonal miRNAs. It was shown that within a certain concentration range, the presence of axonal miRNAs facilitated axonal regeneration. Furthermore, when combined with methylprednisolone, axonal miRNAs reduces the incidence of cyst formation. An enhancement in motor function recovery was observed by the augmentation of axonal miRNAs concentration. Hence, utilization of methylprednisolone in conjunction with material scaffolds and axonal miRNAs represents a viable approach for the treatment of SCI (Zhang et al., 2021).

#### 4.4.2 Thermal electroactive hydrogel combined with electrical stimulation for tissue recovery

As a way to treat SCI, researchers have examined combining electroactive hydrogel (TPEH) with nerve growth factor (NGF) and functional ES. This combined treatment aims to facilitate tissue repair by providing sustained and controlled release of NGF while simultaneously stimulating the affected area with ES. The presence of NGF in TPEH hydrogel promotes differentiation of neural stem cells, and the application of ES further enhances this effect. Following a SCI, there is a disruption in electrical signaling and a decrease in the presence of NGF (Wang et al., 2019). Both electrical impulses and NGF play crucial roles in facilitating the development, migration, and differentiation of neurons (Mahdian et al., 2023). ES is frequently employed in the therapeutic management of SCI. The role of ES extends beyond the preservation of muscle size and the prevention of denervated muscle atrophy. It also plays a crucial role in regulating neuronal development and differentiation (Calvert et al., 2019; Batty et al., 2020). The compound TPEH has been observed to enhance the process of neuronal development while simultaneously inhibiting the differentiation of astrocytes and the creation of scar tissue in *in vitro* experiments. The introduction of hydrogel in combination with ES in rats suffering from SCI resulted in the activation of endogenous neurogenesis and facilitated the restoration of impaired functionality. In summary, the utilization of electroactive hydrogels included with NGF in conjunction with ES exhibits promising potential for the management of SCI in the foreseeable future.

## 5 Conclusion

Here we provided a comprehensive overview of SCI therapy that encompassed therapies utilizing various cellular components, cytokines, and materials. A variety of glial cells, inflammatory cells, and growth factors are involved in the SCI process, and understanding their roles and principles can be used to treat SCI and to promote tissue recovery. In order to enhance the protection of axons and synapses and facilitate repair of the spinal cord following SCI, it is imperative to address the detrimental effects of secondary injury resulting from the inflammatory response. This includes mitigating nerve cell death, axonal breakage, and the degradation of myelin sheaths, among other factors. By effectively preventing or minimizing secondary damage to neural tissues, the overall difficulty of repairing the spinal cord can be significantly reduced. Current data shows that tissue healing can be facilitated by transplantation of foreign stem cells, which undergo proliferation and differentiation to generate particular nerve cells. Furthermore, the modulation of scar formation and inhibitory biomolecules may potentially mitigate the demarcation between healthy and pathological tissues, thereby facilitating the infiltration of nascent axons across the void for effective signal transmission (Li et al., 2021). Currently, microenvironmental modulation biomaterials are promising and show significant advantages over traditional materials for SCI treatment. There are a variety of biomaterials used in the treatment of SCI, including hydrogels, nanoparticles, polymers, and fibrous scaffolds, each of which has its own advantages. Designing biomaterials for the treatment of SCI requires the selection of appropriate materials that meet specific functional and compositional requirements. In order to meet these requirements, a variety of materials are often required, including nano-enzymes, conductive polymers, and ROS-containing scavenging structures, as well as biocompatibilities. Adequate physical support is also necessary to provide the conditions for cellular adherence, proliferation, and differentiation, as well as the creation of a favorable microenvironment for cellular growth (Luo et al., 2024). Biomaterials utilized in the context of SCI therapy thus must satisfy a diverse array of functional requirements. The three key requirements for an effective biomaterial for tissue engineering are: (1) the capacity to adhere to cells, enabling their survival and proliferation at the site of the lesion; (2) a specific internal structure that facilitates cell seeding and promotes axon-oriented growth through linear channels, establishing robust connections between axons; and (3) the ability to gradually release bioactive molecules, ensuring sustained effectiveness over an extended duration for the cells.

Based on the aforementioned information, it is evident that cells, cytokines, and material scaffolds play significant roles in determining the effectiveness of SCI therapies. However, it is crucial to note that the viability of transplanted cells at the lesion of SCI is notably limited. Numerous studies have substantiated that the utilization of biomaterials for cell transplantation yields superior therapeutic outcomes compared to cell transplantation in isolation. Using biomaterials makes it easier to reduce inflammatory responses and increase the number of astrocytes. This helps regenerate axons and direct their growth in the desired location, improving connections at the injury site. Consequently, treatment of SCI with biomaterials contributes to the improvement of tissue and functional recovery (Giorgi et al., 2024). When different types of cells are combined with growth factors, the interfaces between the biomaterial and the surrounding tissue becomes more stable, encouraging axonal regeneration. In some experimental settings, the integration of medicines with biomaterials has been explored, resulting in the development of biomaterial scaffolds capable of controlled and sustained drug release. These scaffolds function as growth promoters for cells that are transplanted within the scaffolds. With the accelerated development of tissue engineering and regenerative medicine, it is widely believed that a wide range of treatments for SCI will become available, giving people with these injuries more reason to be hopeful.

## Author contributions

YG: Writing – original draft. YuW: Writing – review & editing. YaW: Writing – review & editing. SL: Writing – review & editing.
